# Delineating associations of progressive pleuroparenchymal fibroelastosis in patients with pulmonary fibrosis

**DOI:** 10.1183/23120541.00637-2022

**Published:** 2023-03-27

**Authors:** Eyjolfur Gudmundsson, An Zhao, Nesrin Mogulkoc, Frouke van Beek, Tinne Goos, Christopher J. Brereton, Marcel Veltkamp, Robert Chapman, Hendrik W. van Es, Helen Garthwaite, Bahareh Gholipour, Melissa Heightman, Arjun Nair, Katarina Pontoppidan, Recep Savas, Asia Ahmed, Marie Vermant, Omer Unat, Alex Procter, Laurens De Sadeleer, Emma Denneny, Timothy Wallis, Mark Duncan, Magali Taylor, Stijn Verleden, Sam M. Janes, Daniel C. Alexander, Athol U. Wells, Joanna Porter, Mark G. Jones, Iain Stewart, Coline H.M. van Moorsel, Wim Wuyts, Joseph Jacob

**Affiliations:** 1 Centre for Medical Image Computing, Department of Computer Science, UCL, London, UK; 2 Department of Respiratory Medicine, Ege University Hospital, Izmir, Turkey; 3 Interstitial Lung Diseases Center of Excellence, Department of Pulmonology, St Antonius Hospital, Nieuwegein, The Netherlands; 4 BREATHE, Department of Chronic Diseases and Metabolism, KU Leuven, Leuven, Belgium; 5 Department of Respiratory Diseases, University Hospitals Leuven, Leuven, Belgium; 6 NIHR Southampton Biomedical Research Centre and Clinical and Experimental Sciences, University of Southampton, Southampton, UK; 7 Division of Heart and Lungs, University Medical Center, Utrecht, The Netherlands; 8 Interstitial Lung Disease Service, Department of Respiratory Medicine, University College London Hospitals NHS Foundation Trust, London, UK; 9 Department of Radiology, St Antonius Hospital, Nieuwegein, The Netherlands; 10 Royal Free University Hospital NHS Foundation Trust, London, UK; 11 Department of Radiology, University College London Hospitals NHS Foundation Trust, London, UK; 12 Department of Radiology, Ege University Hospital, Izmir, Turkey; 13 Department of Respiratory Medicine, University of Antwerp, Antwerp, Belgium; 14 Lungs for Living Research Centre, UCL, London, UK; 15 Interstitial Lung Disease Unit, Royal Brompton Hospital, Imperial College, London, UK; 16 National Heart and Lung Institute, Imperial College London, London, UK

## Abstract

**Background:**

Computer quantification of baseline computed tomography (CT) radiological pleuroparenchymal fibroelastosis (PPFE) associates with mortality in idiopathic pulmonary fibrosis (IPF). We examined mortality associations of longitudinal change in computer-quantified PPFE-like lesions in IPF and fibrotic hypersensitivity pneumonitis (FHP).

**Methods:**

Two CT scans 6–36 months apart were retrospectively examined in one IPF (n=414) and one FHP population (n=98). Annualised change in computerised upper-zone pleural surface area comprising radiological PPFE-like lesions (Δ-PPFE) was calculated. Δ-PPFE >1.25% defined progressive PPFE above scan noise. Mixed-effects models evaluated Δ-PPFE against change in visual CT interstitial lung disease (ILD) extent and annualised forced vital capacity (FVC) decline. Multivariable models were adjusted for age, sex, smoking history, baseline emphysema presence, antifibrotic use and diffusion capacity of the lung for carbon monoxide. Mortality analyses further adjusted for baseline presence of clinically important PPFE-like lesions and ILD change.

**Results:**

Δ-PPFE associated weakly with ILD and FVC change. 22–26% of IPF and FHP cohorts demonstrated progressive PPFE-like lesions which independently associated with mortality in the IPF cohort (hazard ratio 1.25, 95% CI 1.16–1.34, p<0.0001) and the FHP cohort (hazard ratio 1.16, 95% CI 1.00–1.35, p=0.045).

**Interpretation:**

Progression of PPFE-like lesions independently associates with mortality in IPF and FHP but does not associate strongly with measures of fibrosis progression.

## Abstract 2

**Computerised pleuroparenchymal fibroelastosis (PPFE) progression was found to associate with mortality in idiopathic pulmonary fibrosis and fibrotic hypersensitivity pneumonitis but PPFE did not correlate strongly with measures of fibrosis progression** https://bit.ly/3FKYQn7

## Introduction

Idiopathic pulmonary fibrosis (IPF) is a progressive lung disease characterised by lower zone predominant honeycomb cysts and traction bronchiectasis [1] on computed tomography (CT) imaging [2]. IPF can show a variable disease course [3]. Quantifying disease progression on imaging is important and has primarily involved visual semi-quantification of changes in CT extents of honeycomb cysts, reticulation, ground glass opacities and traction bronchiectasis [4], patterns reflecting pulmonary sequelae of fibrotic damage. Yet estimation of serial CT change in these patterns have shown limited correlations with measures of disease progression [5–7] (change in forced vital capacity (FVC)) and variable correlation with mortality in IPF patients [7].

Computational analysis of CT imaging can identify alternative CT patterns such as vessel-related structures [8–10] which associate with mortality in patients with IPF. Exploration of novel imaging features has also delineated patterns such as pleuroparenchymal fibroelastosis (PPFE) which do not directly result from the fibrotic process, but which may influence patient survival [11]. PPFE is characterised by dense triangular pleurally based opacities occurring in the upper lobes on CT [12]. PPFE scored visually on a single baseline CT has been shown to associate with reduced survival time in IPF patients [11, 13] and other fibrosing lung diseases [14, 15]. Computer quantitation of baseline upper lobe PPFE-like lesions (incidence 25–36%) was shown to associate with mortality in IPF independent of baseline disease severity (measured by either FVC, CT interstitial lung disease (ILD) extent or diffusion capacity of the lung for carbon monoxide (*D*_LCO_)) and identified more patients with a poor outcome than equivalent semi-quantitative visual CT analysis [11]. PPFE-like lesions scored by computer did not associate with baseline measures of IPF-related fibrosis on univariable or multivariable analyses, suggesting that PPFE-related damage might represent injury occurring independent to IPF-related lung fibrosis [11].

Careful delineation of patients with clinically meaningful progressive PPFE-like lesions using computational analysis of time-series CTs may highlight a lung fibrosis endotype benefiting from alternative management strategies [16]. Sensitive quantification of progressive PPFE could also evaluate treatment response in future trials of therapies targeting PPFE and/or progressive fibrotic phenotypes [17] where PPFE change might influence outcome measures. Our current study therefore aimed to delineate in IPF and fibrotic hypersensitivity pneumonitis (FHP) populations the prevalence and prognostic impact of progressive PPFE-like lesions. We also examined whether PPFE change associated with other measures of disease progression in IPF.

## Material and methods

### Study subjects and clinical information

**FIGURE 1:**
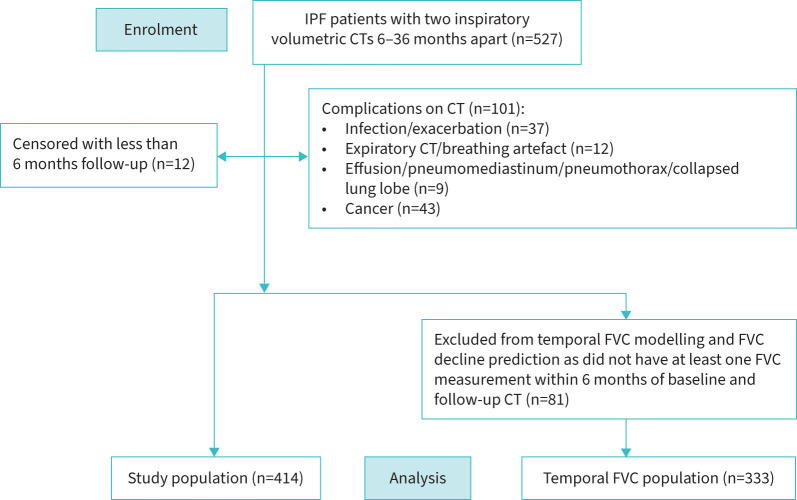
CONSORT diagram showing exclusions for IPF patients in the study. IPF: idiopathic pulmonary fibrosis; CT: computed tomography; FVC: forced vital capacity.

Patients with a multidisciplinary team diagnosis of IPF or FHP with two volumetric CT examinations separated by 6–36 months were identified from five medical centres (Ege University Hospital, Izmir, Turkey; St Antonius Hospital, Nieuwegein, the Netherlands; University Hospital Southampton NHS Foundation Trust, UK; University College London Hospitals NHS Foundation Trust, UK; University Hospitals Leuven, Belgium) (supplementary table S1). CONSORT diagrams for the two study populations are shown in figure 1 and supplementary figure S1; patient demographics for patients included in the study are shown in table 1. Approval for this retrospective study of clinically indicated pulmonary function and CT data was obtained from the local research ethics committees and Leeds East Research Ethics Committee: 20/YH/0120.

**TABLE 1:** Patient demographics, pulmonary function indices and visual and computer-based scores of ILD and PPFE severity for IPF and FHP patients in the study

**Variable**	**IPF cohort**	**FHP cohort**	**p-value**
**Patients n**	414	98	
**Baseline age years, median (range)**	69 (32–95)	64 (28–85)	0.0001
**Male/female %**	24.2/75.8	62.2/37.8	<0.0001
**Survival (alive/dead) %**	44.4/55.6	54.1/45.9	0.11
**Follow-up years, median (range)**	2.2 (0.0–9.0)	2.7 (0.0–12.0)	0.013
**Time between CT scans years, median (range)**	1.1 (0.5–3.0)	1.1 (0.5–2.9)	0.81
**Never-/ever-smokers %**	30.7/69.3	50.0/50.0	0.0005
**Antifibrotic (never/ever) %**	30.7/69.3		
**Baseline FVC % predicted**	81.3±19.7	64.2±19.6	<0.0001
**Baseline *D***_**LCO**_ **% predicted**	48.8±15.9	50.5±16.8	0.44
**Baseline emphysema (absent/present) %**	32.4/67.6	69.4/30.6	<0.0001
**Baseline ILD extent %**	39.0±12.3	33.3±14.0	0.0003
**Δ-ILD %/year**	7.7±8.7	4.0±5.6	<0.0001
**Baseline PPFE extent %**	2.0±2.4	1.9±2.3	0.74
**Δ-PPFE %/year**	0.8±2.0	0.8±2.4	0.93
**Clinically important baseline PPFE prevalence %**	29.5	26.5	0.65
**Progressive PPFE prevalence %**	21.5	25.5	0.47
**Δ-PPFE-adj in progressive PPFE patients %/year**	2.3±2.7	2.4±3.3	0.86

Pulmonary function indices, ILD extent and PPFE scores are described as mean±sd. Clinically important PPFE at baseline was defined as baseline PPFE extent >2.5%. Progressive PPFE was defined as Δ-PPFE >1.25%/year. ILD: interstitial lung disease; PPFE: pleuroparenchymal fibroelastosis; IPF: idiopathic pulmonary fibrosis; FHP: fibrotic hypersensitivity pneumonitis; CT: computed tomography; FVC: forced vital capacity; *D*_LCO_: diffusing capacity of the lung for carbon monoxide; Δ-ILD: annualised change in ILD extent between CT scans; Δ-PPFE: annualised change in computerised upper-zone PPFE between scans; Δ-PPFE-adj: Δ-PPFE above scan noise.

### Visual CT evaluation

A subspecialist radiologist (J. Jacob) with 14 years of thoracic imaging experience determined lobar percentages of ILD (sum of ground glass density, reticulation, traction bronchiectasis volume and honeycomb cysts, averaged across six lobes [11]) on two timepoint CTs, and emphysema presence (absent/present) on baseline CTs of all IPF and FHP patients. Change in CT ILD extent was annualised (“Δ-ILD”). The radiologist was blinded to outcome data when visually evaluating the CTs of the study.

### Computer-based CT evaluation

Computerised quantification of the percentage of visceral pleural surface (most peripheral 3 lung surface pixels) affected by radiological PPFE-like lesions was obtained on CT pairs of IPF and FHP patients as previously described [11]. PPFE-like lesions were only quantified in the upper zones (region extending from carina to 5 mm below lung apices – thereby avoiding capturing an apical cap) which approximated the upper lobes, where radiological PPFE is most typically found. A 2.5% threshold of PPFE extent on baseline imaging, derived in a previous IPF study [11], delineated clinically important PPFE at baseline for IPF and FHP cohorts.

The annualised change in computerised upper-zone extent of PPFE-like lesions between scans (“Δ-PPFE”) was calculated as the difference in computerised PPFE between the baseline and follow-up CTs, divided by the scan interval in years. It is important to account for the contribution of noise to the estimation of PPFE change between CT scans. Noise can occur between CT timepoints due to differences (for example) in CT acquisition parameters including scanner model and reconstruction algorithm variability, in the level of patient inspiration, and in patient positioning. Δ-PPFE of 1.25% or more of the pleural surface area was used to identify patients with morphologically definitive “progressive” radiological PPFE-like lesions. The estimation of noise contained within the longitudinal CT imaging was determined by calculating one half of the standard deviation of baseline PPFE in the derivation IPF cohort [11], which corresponds to a moderate effect size. This method has previously been suggested to determine the minimal clinically important difference in biomarker studies in IPF [18–20]. A continuous variable (“Δ-PPFE-adj”) reflecting definitive change in extent of PPFE-like lesions above scan noise was created by subtracting 1.25% from Δ-PPFE values of progressive radiological PPFE patients and setting Δ-PPFE-adj values to 0% for non-progressive patients.

### Modelling strategy

Linear mixed-effects (LME) regression analyses, with a single fixed effect and a random intercept for each centre, investigated the association between longitudinal change of ILD extent and PPFE extent, and baseline ILD, PPFE, *D*_LCO_ % predicted, and FVC % predicted in both cohorts. LME models with multiple fixed effects were also used to investigate the association between change in FVC and Δ-PPFE. The temporal trajectories of FVC measurements were modelled between baseline and follow-up CTs separately for each cohort, with a random intercept for each centre and each subject, and with a random slope for each subject. These models included fixed effects of age at baseline, patient sex, smoking status (never/ever), baseline emphysema presence, baseline FVC % predicted, study time and Δ-PPFE. In the IPF cohort, these models also included fixed effects of antifibrotic use across follow-up (never/ever).

Across all LME models for FVC, patients who did not have at least two absolute FVC measurements (one within 6 months of baseline CT and another within 6 months of follow-up CT) were excluded (IPF: 81 excluded out of 414; FHP: 20 excluded out of 98). Modelled FVC measurements were restricted to 6 months prior to the baseline CT of each patient and 6 months after the follow-up CT of each patient, to ensure that FVC trajectories were representative of the development of disease between the scans.

Univariable and multivariable Cox regression analyses explored determinants of mortality, with a single frailty variable for centre to adjust for mean differences between patient centres within each cohort. Entry time for survival analysis was taken as the date of second CT. All multivariable mortality models were adjusted for patient age at baseline, patient sex, smoking status (never/ever), baseline emphysema presence (absent/present), Δ-ILD, clinically important PPFE at baseline (no/yes) and *D*_LCO_ % predicted. Antifibrotic use (never/ever) adjustments were used in the IPF cohort only.

Baseline *D*_LCO_ % predicted and baseline FVC % predicted were considered if available within 3 months of baseline CT. Missing baseline *D*_LCO_ % predicted and baseline FVC % predicted were imputed and considered missing at random (details in online supplementary material).

### Statistical analysis

Data are presented as patient proportions (%) or mean±sd or medians (with range of values), as appropriate. Differences in categorical variables were assessed using the Chi-squared test. Differences in medians of continuous variables were assessed using the two-sided Mann–Whitney U-test. Differences in means of continuous variables were assessed using the two-sided t-test. In three-group comparisons, a Kruskal–Wallis rank sum test evaluated differences in medians and a one-way ANOVA evaluated differences in means. A p-value <0.05 was considered significant across all analyses. Multivariable linear models were tested for heteroscedasticity using the studentised Breusch–Pagan test [21]. The Concordance index (C-index) compared the goodness of fit of Cox regression models [22]. R^2^ values reported for LME models are the “marginal” R^2^, which describes the proportion of variance explained by fixed factor(s) alone [23]. Bootstrapping with 500 iterations was used to estimate sampling distributions of the C-index. Kaplan–Meier curves were truncated at 5 years. LME model analyses, Cox regression and Kaplan–Meier analyses, and multiple imputations were performed with the lme4, survival and mice packages in R, respectively (version 4.1.1 with RStudio version 1.4.1717; RStudio, Boston, MA, USA).

## Results

### Baseline data

Demographic data, baseline pulmonary function tests, and mean visual ILD extent and computerised PPFE scores for the IPF cohort (n=414) and the FHP cohort (n=98) are shown in table 1. Baseline characteristics of IPF patients and FHP patients excluded from the study are shown in supplementary tables S2 and S3, respectively.

### Computerised PPFE extent associations

**FIGURE 2:**
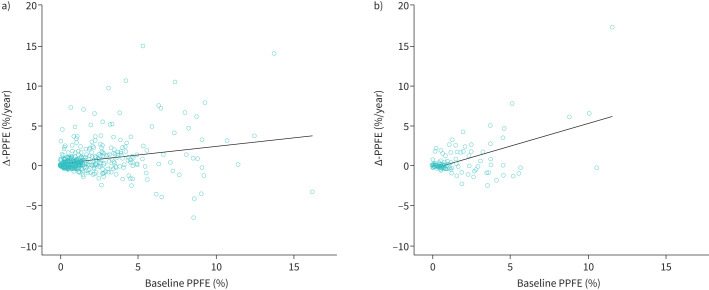
Association between Δ-PPFE and baseline PPFE extent in a) the IPF cohort (effect: 0.21%/year, 95% CI 0.13–0.29%/year, p<0.0001, R^2^=0.06) and b) the FHP cohort (effect: 0.56%/year, 95% CI 0.38–0.75%/year, p<0.0001, R^2^=0.28). PPFE: pleuroparenchymal fibroelastosis; Δ-PPFE: annualised change in computerised upper-zone PPFE between scans; IPF: idiopathic pulmonary fibrosis; FHP: fibrotic hypersensitivity pneumonitis.

The prevalence of clinically important PPFE (*i.e.*, PPFE extent >2.5%) on baseline imaging was 29.5% in the IPF cohort and 26.5% in the FHP cohort (table 1). Baseline computerised PPFE extent weakly associated with Δ-PPFE in the IPF cohort but slightly more strongly in the FHP cohort (figure 2 and supplementary table S4). Baseline PPFE weakly associated with Δ-ILD in univariable models in the IPF cohort only (supplementary figure S2 and table S4).

Δ-PPFE weakly associated with Δ-ILD in the IPF cohort but not the FHP cohort (supplementary figure S3 and table S4). Δ-PPFE weakly associated with baseline *D*_LCO_ % and baseline FVC % in multivariable models in both cohorts (supplementary tables S4 and S5 and figures S4 and S5). Comparisons between baseline ILD and Δ-ILD and Δ-PPFE are shown in supplementary figures S6 and S7.

### PPFE change and FVC decline

Demographic data, baseline pulmonary function tests, and mean visual ILD extent and computerised PPFE scores for patients included and excluded from FVC modelling in the IPF cohort and the FHP cohort are shown in supplementary tables S6 and S7, respectively. Δ-PPFE weakly associated with FVC change in univariable models in the IPF cohort only (−0.13 L·year^−1^, 95% CI −0.18– −0.08 L·year^−1^, p<0.0001, R^2^=0.07) and in multivariable models in the IPF cohort only (effect: −0.09 L·year^−1^, 95% CI −0.13– −0.05 L·year^−1^, p<0.0001, R^2^=0.34) (supplementary tables S8 and S9). Results were maintained in non-imputed models in both cohorts (supplementary table S10).

### PPFE change associations with mortality

In univariable Cox regression models in the IPF cohort, covariates significantly associated with mortality included: baseline *D*_LCO_ % predicted, baseline FVC % predicted, baseline ILD extent, baseline PPFE extent, presence of clinically important PPFE at baseline, Δ-PPFE and Δ-PPFE-adj (supplementary table S11). In the FHP cohort, covariates significantly associated with mortality in univariable Cox regression models included: patient age at baseline, baseline *D*_LCO_ % predicted, baseline ILD extent, baseline PPFE extent, Δ-PPFE and Δ-PPFE-adj (supplementary table S11).

In multivariable Cox regression models, Δ-PPFE was significantly associated with mortality in the IPF cohort (hazard ratio (HR) 1.20, 95% CI 1.13–1.28, p<0.0001) and the FHP cohort (HR 1.18, 95% CI 1.05–1.34, p=0.008) (supplementary table S12). Results were maintained in non-imputed models in both cohorts (supplementary table S13). Multivariable Cox regression models without adjustment for Δ-PPFE are shown in supplementary table S14.

### PPFE progression above scan noise

**FIGURE 3:**
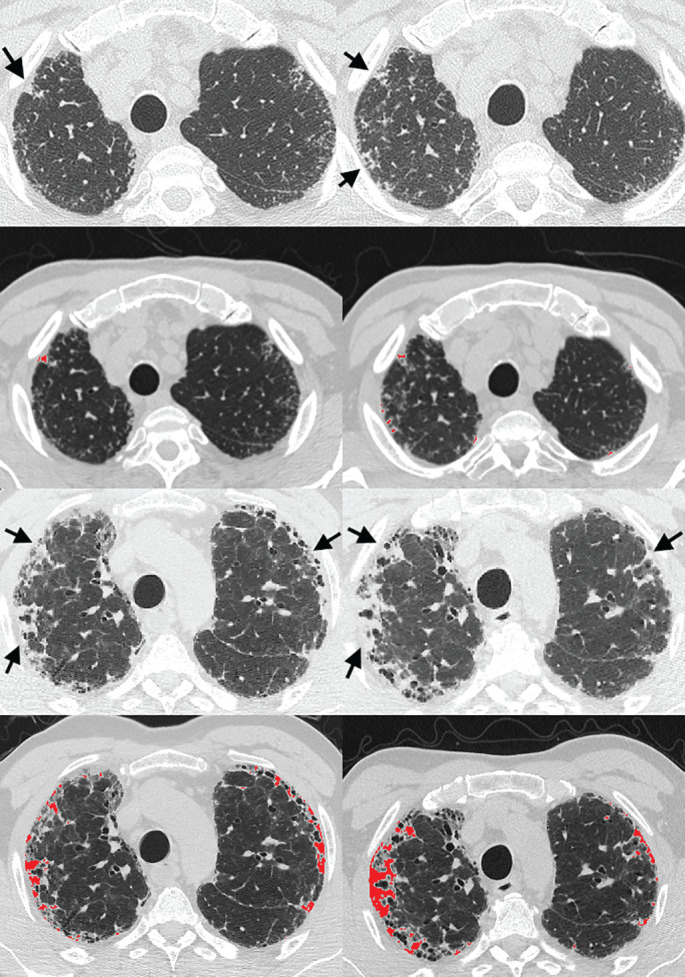
Visual characteristics of progressive pleuroparenchymal fibroelastosis (PPFE). Baseline (left column) and follow-up (right column) computed tomography (CT) scans in patients diagnosed with idiopathic pulmonary fibrosis by multidisciplinary team. The top two rows show axial CT images of the upper zones taken 13 months apart in a 73-year-old male with normal baseline forced vital capacity (FVC) (83%) and diffusion capacity of the lung for carbon monoxide (*D*_LCO_) (74%). Arrows (top row) show areas of PPFE increasing in extent on the second CT, also highlighted by image overlays of PPFE regions (second row). The bottom two rows show axial CT images taken 9 months apart in a 54-year-old male with abnormal baseline FVC (46%) and *D*_LCO_ (35%). Arrows (third row) show more extensive PPFE proliferating dramatically on the second CT, again highlighted on image overlays of PPFE regions (bottom row).

89 out of 414 (21%) patients in the IPF cohort and 25 out of 98 (26%) patients in the FHP cohort had progressive radiological PPFE as determined by Δ-PPFE >1.25%/year (figure 3). Demographic data, baseline pulmonary function tests, and mean visual ILD extent and computerised PPFE scores for non-progressive PPFE patients without clinically important PPFE at baseline, non-progressive PPFE patients with clinically important PPFE at baseline, and progressive PPFE patients are shown in supplementary tables S15 and S16.

**FIGURE 4:**
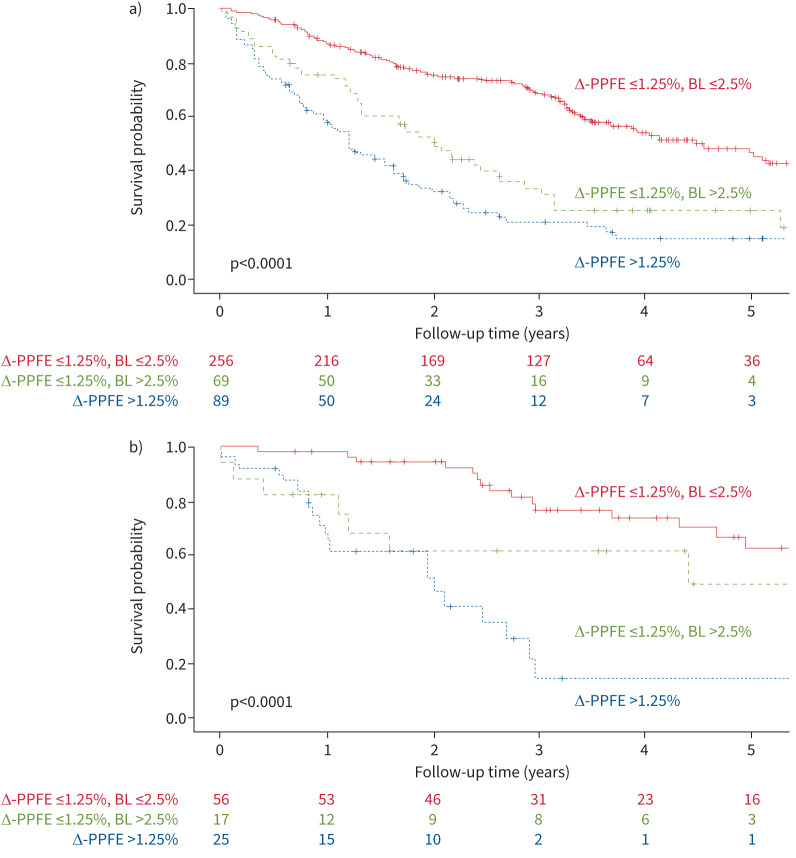
The impact of progressive PPFE and clinically important PPFE at baseline on survival in IPF and FHP patients. Kaplan–Meier survival curves for: 1) patients without clinically important PPFE at baseline and without progressive PPFE (“Δ-PPFE ≤1.25%, BL ≤2.5%”); 2) patients with clinically important PPFE at baseline and without progressive PPFE (“Δ-PPFE ≤1.25%, BL >2.5%”); and 3) patients with progressive PPFE (“Δ-PPFE >1.25%”) in a) the IPF cohort and b) the FHP cohort. Progressive PPFE was defined as patients with Δ-PPFE >1.25%/year change in pleural surface area. Clinically important PPFE at baseline was defined as PPFE baseline extent >2.5%. Survival curves were truncated at 5 years. Data below each plot show number of patients at risk at 1-year intervals. p-values are based on a log-rank test of differences in the survival curves of each plot. IPF: idiopathic pulmonary fibrosis; FHP: fibrotic hypersensitivity pneumonitis; PPFE: pleuroparenchymal fibroelastosis; Δ-PPFE: annualised change in computerised upper-zone PPFE between scans; BL: baseline.

Definitive PPFE change above scan noise (Δ-PPFE-adj) was independently associated with mortality in multivariable Cox regression models in the IPF cohort (HR 1.25, 95% CI 1.16–1.34, p<0.0001) and the FHP cohort (HR 1.16, 95% CI 1.00–1.35, p=0.045) (table 2) regardless of the degree of ILD progression or the presence of clinically important PPFE at baseline. Results were maintained in non-imputed models and in models not adjusted for baseline presence of clinically important PPFE (supplementary tables S17 and S18). Sensitivity analyses investigating noise threshold values in the range 0.5%/year to 1.5%/year showed maintained results (supplementary table S19). Kaplan–Meier analyses reflected the poor survival in patients with progressive PPFE (figure 4).

**TABLE 2:** Association of Δ-PPFE-adj with mortality in multivariable Cox regression models in the IPF cohort and in the FHP cohort

**Variable**	**Hazard ratio**	**95% confidence interval**	**p-value**	**Model C-index**
**IPF**				
** **Baseline age years	1.00	0.99–1.02	0.73	0.75
** **Male sex	1.46	1.00–2.14	0.047	
** **Ever-smoker	1.22	0.87–1.71	0.25	
** **Baseline emphysema (absent/present)	0.93	0.67–1.31	0.69	
** **Antifibrotic treatment (never/ever)	0.72	0.54–0.97	0.033	
** **Δ-ILD %/year	1.01	0.98–1.03	0.62	
** **Baseline PPFE extent >2.5%	1.72	1.27–2.33	0.0006	
** **Baseline *D*_LCO_ % predicted	0.96	0.94–0.97	<0.0001	
** **Δ-PPFE-adj %/year	1.25	1.16–1.34	<0.0001	
**FHP**				
** **Baseline age years	1.08	1.03–1.13	0.004	0.79
** **Male sex	1.02	0.41–2.52	0.97	
** **Ever-smoker	1.75	0.63–4.86	0.27	
** **Baseline emphysema (absent/present)	0.71	0.29–1.74	0.44	
** **Δ-ILD %/year	1.13	1.04–1.23	0.004	
** **Baseline PPFE extent >2.5%	2.11	0.89–5.00	0.086	
** **Baseline *D*_LCO_ % predicted	0.96	0.93–0.99	0.014	
** **Δ-PPFE-adj %/year	1.16	1.00–1.35	0.045	

Models in all cohorts were adjusted for patient age, sex, smoking history (never/ever), emphysema presence at baseline, clinically important PPFE at baseline (baseline PPFE >2.5% upper-zone pleural surface area), baseline *D*_LCO_ % predicted, annualised change in interstitial lung disease extent (Δ-ILD) and Δ-PPFE-adj. Models in the IPF cohort were also adjusted for antifibrotic treatment (never/ever). Δ-PPFE-adj: annualised change in computerised upper-zone PPFE between scans above scan noise; IPF: idiopathic pulmonary fibrosis; FHP: fibrotic hypersensitivity pneumonitis; PPFE: pleuroparenchymal fibroelastosis; *D*_LCO_: diffusing capacity of the lung for carbon monoxide.

## Discussion

In our study of computerised quantitation of radiological PPFE change we demonstrate that in patients with IPF and FHP, worsening computerised PPFE independently associates with increased patient mortality with similar effect sizes seen in two separate patient populations. Limited associations were seen between PPFE worsening and measures used to estimate disease progression in IPF and FHP (radiological ILD progression and FVC decline) suggesting that PPFE progression occurs independently of established fibrotic pathways. When evaluating morphologically important PPFE change, over 20% of patients in the IPF and FHP cohorts demonstrated progressive PPFE.

Radiological PPFE on CT has been well characterised over the last 10 years following detailed histopathological–radiological correlative studies [24–26]. PPFE quantification has primarily been attempted using crude categorical visual scales of CT disease extent [11, 14], but computer quantitation can improve identification of PPFE patients with a poor prognosis [11]. In the current study the high prevalence of PPFE noted in FHP [15] and similar survival between FHP and IPF patients [27, 28] underpinned the rationale for extending our analysis to a multicentred FHP cohort. We show that across two cohorts of patients with fibrosing lung disease, similar proportions of patients demonstrated a progressive PPFE phenotype. The disassociation between PPFE progression and ILD progression in the current study (as delineated by CT change in visual ILD scores or FVC decline) matched disassociation between baseline PPFE extent and measures of IPF severity in our previous study [11].

While PPFE has been increasingly reported in patients with underlying lung fibrosis [11, 14], PPFE has also been identified in the setting of bone marrow [25, 29] and lung transplantation [30–32] recipients, patients exposed to dusts [33] and as a long-term sequela of patients receiving chemotherapeutic agents [34]. Hypotheses for potential causes of PPFE might include occult infectious agents, excessive reaction to recurrent pulmonary infections in patients with pre-existing immune dysregulation or a manifestation of a pulmonary malignancy. Associations seen in patients with PPFE include genetic predispositions (telomere-related gene mutations [35, 36]), recurrent pulmonary infections [24] and ischaemia in apical lung vessels [30, 37, 38]. Despite the high prevalence of PPFE reported in patients with interstitial fibrosis, the location of PPFE in the lung apices, its occurrence in patients without fibrosis and specifically the lack of major association between PPFE progression and measures of fibrosis progression support our contention [11] that PPFE occurring in fibrosing lung diseases represents a distinct disease endotype.

The increased mortality seen in patients with progressive PPFE may reflect the replacement of upper-zone lung tissue in patients with IPF/FHP with an elastotic process. The lung in the upper zones is often spared in IPF, and its loss may disproportionately impact gas exchange in patients where the middle/lower zones demonstrate airspace and vascular destruction. It has also been observed in studies using corrosion casting of the lung microvasculature that extensive intussusceptive angiogenesis occurs in areas of PPFE [39]. It is therefore possible that as PPFE proliferates, there may be accentuation in ventilation–perfusion mismatches which in turn further exacerbate hypoxia in patients with concomitant fibrosing lung disease.

There remains an urgent need to invest more resources to examine specific treatments that can target PPFE in the subgroups of patients with IPF and FHP. Application of computer tools to baseline CT data [11] can aid in cohort enrichment when recruiting patients to therapeutic trials for PPFE, which can in turn improve the power of clinical trials. Therapeutic trials in IPF have been constrained by a lack of reliable end-points, which necessitate larger sample sizes and therefore more expensive trials. Our current study emphasises the potential for computer-based delineation of PPFE progression to act as a drug trial end-point when determining treatment response.

There were several limitations to the current study. Reasons for performing longitudinal imaging in IPF and FHP patients can be varied. Disproportionately, imaging is repeated following clinical deterioration. Consequently, patients with acute exacerbations, infections, pneumothoraces [25, 40] and pneumomediastinum, which occur with increased frequency in PPFE, were not infrequent in our study cohorts (figure 1, supplementary figure S1). These patients and those with coexisting lung malignancies were excluded from the current analysis to avoid non-PPFE pathology being mistakenly characterised as PPFE by the computer. We may therefore have underestimated the prevalence of progressive PPFE occurring in IPF and FHP. In addition, we did not have detailed information in the study population on the use of immunomodulatory therapies, which could have influenced the progression of PPFE.

Through a retrospective analysis of non-protocolised scans, our study demonstrated a strong mortality signal in real-world multicentred noisy data. While we adjusted our analyses to account for biases between study centres through mixed-effects models and frailty Cox models, we also tried to delineate measurement noise associated with quantitative analysis of longitudinal CT imaging. As it is only necessary to detect a digital signature equivalent to PPFE on one single voxel out of the many millions of lung voxels present on a single CT, some measure of PPFE will invariably be detected by computational CT analysis. However not all PPFE or PPFE change detected by a computer is real, *e.g.*, PPFE change could be artificially inflated by a poor inspiratory effort on a second timepoint CT. We estimated the degree of noise from longitudinal CT analysis of PPFE change as 50% of the standard deviation of PPFE seen at baseline, in accordance with similar prior work in IPF. The similar effect size of our adjusted PPFE quantitation on mortality analysis across the two study cohorts reinforces our belief that our estimation of noise is appropriate and has clinical utility.

In conclusion, our study highlights the independent deleterious prognostic effect of worsening computerised PPFE-like lesions in patients with IPF and FHP. PPFE progression only associated weakly with measures of ILD progression in IPF suggesting that the distinct disease trajectories for ILD and PPFE may represent separate pathophysiological pathways. Over 20% of patients in the two study cohorts were identified with a progressive PPFE phenotype which independently associated with mortality. Given the need for new targeted therapies for PPFE, our computer-based quantitation of PPFE could act as a new end-point in randomised clinical trials.

## Supplementary material

10.1183/23120541.00637-2022.Supp1 **Please note:** supplementary material is not edited by the Editorial Office, and is uploaded as it has been supplied by the author.
Supplementary material 00637-2022.SUPPLEMENT
