# Supplementary material for: Delineating associations of progressive pleuroparenchymal fibroelastosis in patients with pulmonary fibrosis
Source: ERJ Open Res. 2023 Mar 27;9(2):00637-2022. doi: 10.1183/23120541.00637-2022 (PMC10052711; doi:10.1183/23120541.00637-2022)
Supplement: Supplementary file 1 [file 00637-2022.SUPPLEMENT.pdf]

Supplementary Appendix

Supplementary Methods

Imputation of missing DLco and FVC values at baseline

Missing values of DLco% predicted and FVC% predicted within 3 months of baseline CT were considered missing at random. These values were imputed in LME models for FVC change and Cox regression models for mortality using the predictive mean matching method of multiple imputation. Multiple imputations were performed with the mice package in R (version 4.1.1 with Rstudio version 1.4.1717, Rstudio, Massachusetts, US). Patient centre, patient age at baseline CT, and patient gender were used as predictor variables in all imputed models, with the first available FVC (l) measurement of each patient also included in all imputed LME models for FVC change. Other predictor variables were determined using the default settings of the built-in quickpred function of the mice package. An indicator whether patient passed away during follow-up and follow-up time (in years) were included as the set of potential predictor variables in imputed Cox regression models for mortality. R^2 and C-index values for imputed models are the median values across all imputed models. 100 imputed models were generated for each analysis.[1] Parameter values were pooled according to Rubin's rules using the pool function of the mice package.

Supplementary Table 1. **Patient cohorts and medical centres.** The IPF cohort comprised patients presenting to Ege University Hospital, Izmir, Turkey between 2008-2015, St Antonius Hospital, Nieuwegein, Netherlands between 2004-2019, University Hospital Southampton NHS Foundation Trust, Southampton, UK between 2013-2015, University College London Hospitals NHS Foundation Trust, London, UK between 2012-2019, and University Hospitals Leuven, Leuven, Belgium between 2012-2017. The FHP cohort consisted of patients presenting to Ege University Hospital, Izmir, Turkey between 2008-2015, University Hospital Southampton NHS Foundation Trust, Southampton, UK between 2013-2015, University College London Hospitals NHS Foundation Trust, London, UK between 2012-2019, and St Antonius Hospital, Nieuwegein, Netherlands between 2007-2019. IPF = idiopathic pulmonary fibrosis, PPFE = pleuroparenchymal fibroelastosis, FHP = fibrotic hypersensitivity pneumonitis.

Cohort	Medical centre	No. patients
IPF cohort (n = 414)	Ege University Hospital, Izmir, Turkey	94
	St Antonius Hospital, Nieuwegein, Netherlands	166
	University Hospital Southampton NHS Foundation Trust, Southampton, UK	24
	University College London Hospitals NHS Foundation Trust, London, UK	80
	University Hospitals Leuven, Leuven, Belgium	50
FHP cohort (n = 98)	Ege University Hospital, Izmir, Turkey	18
	University Hospital Southampton NHS Foundation Trust, Southampton, UK	23
	University College London Hospitals NHS Foundation Trust, London, UK	16
	St Antonius Hospital, Nieuwegein, Netherlands	41

Supplementary Table 2. Demographic data for IPF patients excluded from the study. Baseline demographic data and pulmonary function indices in patients who were excluded from the IPF cohort. Statistical comparisons were made against the patients of the IPF cohort included in the study (Table 1). IPF = idiopathic pulmonary fibrosis, FVC = forced vital capacity, DLco = diffusing capacity for carbon monoxide.

**Variable**	**Excluded IPF (n = 113)**	**p-value**
Median baseline age in years (range)	67 (28 – 97)	0.57
Male / female	83.2% / 16.8%	0.13
Survival (alive / dead)	28.3% / 71.7%	0.003
Median years of follow-up (range)	0.8 (0.0 – 7.6)	<0.0001
Never / ever smokers	12.4% / 87.6%	0.0002
Antifibrotic (never / ever)	44.2% / 55.8%	0.009
FVC% predicted	60.6 +/- 22.6	<0.0001
DLco% predicted	57.9 +/- 25.7	0.004
Median years between CT scans (range)	1.1 (0.0 – 9.4)	0.60

Supplementary Table 3. Demographic data for FHP patients excluded from the study. Baseline demographic data and pulmonary function indices in patients who were excluded from the FHP cohort. Statistical comparisons were made against the patients of the FHP cohort included in the study (Table 1). FHP = fibrotic hypersensitivity pneumonitis, FVC = forced vital capacity, DLco = diffusing capacity for carbon monoxide.

**Variable**	**Excluded FHP (n = 19)**	**p-value**
Median baseline age in years (range)	67 (54 – 80)	0.08
Male / female	42.1% / 57.9%	0.92
Survival (alive / dead)	63.2% / 36.8%	0.63
Median years of follow-up (range)	0.5 (0.0 – 6.2)	0.003
Median years between CT scans (range)	1.0 (0.5 – 2.7)	0.60
Never / ever smokers	78.9% / 21.1%	0.04
FVC% predicted	68.6 +/- 15.7	0.33
DLco% predicted	47.0 +/- 18.5	0.51

Supplementary Table 4. Univariable linear mixed-effects regression analyses between Δ-ILD and baseline PPFE, Δ-PPFE, and baseline ILD extent, and between Δ-PPFE and baseline PPFE, baseline ILD extent, baseline DLco and baseline FVC in IPF patients and FHP patients in the study. Univariable linear mixed-effects regression analyses, adjusted for patient centre as a random intercept, demonstrating relationships between Δ-ILD and i) baseline PPFE, ii) Δ-PPFE, and iii) baseline ILD extent, and between Δ-PPFE and i) baseline PPFE extent, ii) baseline ILD extent, iii) baseline DLco% predicted, and iv) baseline FVC% predicted in the IPF cohorts and the FHP cohort. The marginal R^2 values shown describe only the proportion of variance explained by the fixed effect of each model. IPF = idiopathic pulmonary fibrosis, FHP = fibrotic hypersensitivity pneumonitis, DLco = diffusing capacity for carbon monoxide, FVC = forced vital capacity, ILD = interstitial lung disease, Δ-ILD = annualised change in ILD extent between scans, PPFE = pleuroparenchymal fibroelastosis, Δ-PPFE = annualised change in computerised upper-zone PPFE between scans.

**Cohort**	**Dependent variable**	**Independent variable**	**Effect [%/year]**	**95% Confidence Interval [%/year]**	**p-value**	**Model R^2 value**
IPF	Δ-ILD	Baseline PPFE	0.36	0.01, 0.70	0.043	0.01
		Δ-PPFE	1.05	0.66, 1.44	<0.0001	0.06
		Baseline ILD	-0.15	-0.22, -0.09	<0.0001	0.05
	Δ-PPFE	Baseline PPFE	0.21	0.13, 0.29	<0.0001	0.06
		Baseline ILD	0.02	-0.001, 0.03	0.064	0.01
		Baseline DLco	-0.01	-0.03, 0.0001	0.051	0.01
		Baseline FVC	-0.02	-0.03, -0.01	0.0003	0.04
FHP	Δ-ILD	Baseline PPFE	0.32	-0.17, 0.80	0.20	0.02
		Δ-PPFE	0.37	-0.08, 0.82	0.11	0.03
		Baseline ILD	-0.02	-0.10, 0.06	0.59	0.003
	Δ-PPFE	Baseline PPFE	0.56	0.38, 0.75	<0.0001	0.28
		Baseline ILD	0.03	-0.002, 0.07	0.063	0.04
		Baseline DLco	-0.03	-0.07, 0.003	0.076	0.05
		Baseline FVC	-0.03	-0.06, -0.005	0.023	0.07

Supplementary Table 5. Multivariable linear mixed-effects regression analyses between ∆-PPFE and baseline DLco and baseline FVC, adjusted for patient centre as a random intercept, in IPF patients and FHP patients in the study. Multivariable linear mixed-effects regression analyses, adjusted for patient centre as a random intercept, demonstrating relationships between ∆-PPFE and a) baseline DLco% predicted, b) baseline FVC% predicted in the IPF cohort and the FHP cohort. The marginal R^2 values shown describe only the proportion of variance explained by the fixed effect in each model. IPF = idiopathic pulmonary fibrosis, FHP = fibrotic hypersensitivity pneumonitis, DLco = diffusing capacity for carbon monoxide, FVC = forced vital capacity, PPFE = pleuroparenchymal fibroelastosis, ∆-PPFE = annualised change in computerised upper-zone PPFE between scans. *: model Breusch-Pagan p value < 0.05.

Cohort	Dependent variable	Independent variable	Effect [%/year]	95% Confidence Interval [%/year]	p-value	Model R^2 value
IPF	∆-PPFE	Baseline DLco	-0.02*	-0.03, -0.004	0.01	0.04
		Baseline FVC	-0.02*	-0.03, -0.01	0.0002	0.07
FHP	∆-PPFE	Baseline DLco	-0.09	-0.16, -0.02	0.01	0.18
		Baseline FVC	-0.04	-0.08, -0.007	0.021	0.15

Supplementary Table 6. Demographic data for IPF patients included and excluded from FVC modelling. Baseline demographic data, baseline pulmonary function indices, mean visual ILD extent, and computerised PPFE scores in IPF patients who were included in FVC modelling and IPF patients who were excluded from FVC modelling. Clinically important PPFE at baseline was defined as baseline PPFE extent >2.5%. Progressive PPFE was defined as Δ-PPFE >1.25%/year. IPF = idiopathic pulmonary fibrosis, FVC = forced vital capacity, DLco = diffusing capacity for carbon monoxide, PPFE = pleuroparenchymal fibroelastosis, Δ-PPFE = annualised change in computerised upper-zone PPFE between scans, Δ-PPFE-adj = annualised change in computerised upper-zone PPFE between scans above scan noise.

**Variable**	**IPF patients included in FVC modelling (n = 333)**	**IPF patients excluded from FVC modelling (n = 81)**	**p-value**
Median baseline age in years (range)	69 (32 – 88)	70 (50 – 95)	0.25
Male / female	73.9% / 26.1%	84.0% / 16.0%	0.079
Survival (alive / dead)	46.2% / 53.8%	37.0% / 63.0%	0.17
Median years of follow-up (range)	2.4 (0.1 - 8.2)	1.4 (0.0 - 9.0)	0.0003
Never / ever smokers	32.1% / 67.9%	24.7% / 75.3%	0.24
Antifibrotic (never / ever)	26.7% / 73.3%	46.9% / 53.1%	0.0007
FVC% predicted	81.5 +/- 20.0	79.0 +/- 16.5	0.43
DLco% predicted	48.8 +/- 16.1	49.3 +/- 14.0	0.86
Median years between CT scans (range)	1.1 (0.5 - 3.0)	1.2 (0.5 - 3.0)	0.18
Baseline emphysema (absent/present)	33.6% / 66.4%	27.2% / 72.8%	0.32
Baseline ILD extent (%)	38.7 +/- 12.5	40.5 +/- 11.5	0.21
Δ-ILD (%/year)	7.8 +/- 8.8	7.0 +/- 8.3	0.42
Baseline PPFE extent (%)	2.0 +/- 2.3	2.1 +/- 2.5	0.83
Δ-PPFE (%/year)	0.6 +/- 1.8	1.3 +/- 2.9	0.044
Clinically important baseline PPFE prevalence	30.0%	27.2%	0.71
Progressive PPFE prevalence	19.8%	28.4%	0.13
Δ-PPFE-adj in progressive PPFE patients (%/year)	2.0 +/- 2.3	3.0 +/- 3.8	0.22

Supplementary Table 7. Demographic data for FHP patients included and excluded from FVC modelling. Baseline demographic data, baseline pulmonary function indices, mean visual ILD extent, and computerised PPFE scores in FHP patients who were included in FVC modelling and patients who were excluded from FVC modelling. Clinically important PPFE at baseline was defined as baseline PPFE extent >2.5%. Progressive PPFE was defined as Δ-PPFE >1.25%/year. FHP = fibrotic hypersensitivity pneumonitis, FVC = forced vital capacity, DLco = diffusing capacity for carbon monoxide, PPFE = pleuroparenchymal fibroelastosis, Δ-PPFE = annualised change in computerised upper-zone PPFE between scans, Δ-PPFE-adj = annualised change in computerised upper-zone PPFE between scans above scan noise.

**Variable**	**FHP patients included in FVC modelling (n = 78)**	**FHP patients excluded from FVC modelling (n = 20)**	**p-value**
Median baseline age in years (range)	64 (28 – 85)	63 (40 – 85)	0.97
Male / female	38.5% / 61.5%	35.0% / 65.0%	0.98
Survival (alive / dead)	50.0% / 50.0%	70.0% / 30.0%	0.18
Median years of follow-up (range)	2.7 (0.0 - 10.4)	2.9 (0.0 - 12.0)	0.66
Never / ever smokers	52.6% / 47.4%	40.0% / 60.0%	0.45
FVC% predicted	65.9 +/- 18.9	52.9 +/- 21.0	0.090
DLco% predicted	50.3 +/- 16.6	52.9 +/- 19.3	0.74
Median years between CT scans (range)	1.1 (0.5 - 2.9)	1.3 (0.5 - 2.8)	0.14
Baseline emphysema (absent/present)	73.1% / 26.9%	55.0% / 45.0%	0.20
Baseline ILD extent (%)	33.2 +/- 13.7	33.8 +/- 15.3	0.87
Δ-ILD (%/year)	3.6 +/- 5.6	5.6 +/- 5.2	0.13
Baseline PPFE extent (%)	1.8 +/- 1.9	2.5 +/- 3.3	0.34
Δ-PPFE (%/year)	0.6 +/- 1.7	1.6 +/- 4.2	0.29
Clinically important baseline PPFE prevalence	23.1%	40.0%	0.21
Progressive PPFE prevalence	25.6%	25.0%	1.00
Δ-PPFE-adj in progressive PPFE patients (%/year)	1.6 +/- 1.7	5.5 +/- 6.1	0.23

Supplementary Table 8. **Univariable linear mixed-effects regression analyses between temporal FVC (l) measurements and Δ-PPFE.** Univariable linear mixed-effects regression analyses, adjusted for patient centre as a random intercept and for subject as a random slope and a random intercept, demonstrating relationships between FVC (l) change within a 6-month window of the baseline and follow-up CT scans of each patient and Δ-PPFE in the IPF cohort and the FHP cohort. R^2 values shown are the marginal R^2 describing only the proportion of variance explained by the fixed effect of Δ-PPFE. IPF = idiopathic pulmonary fibrosis, FHP = fibrotic hypersensitivity pneumonitis, FVC = forced vital capacity, PPFE = pleuroparenchymal fibroelastosis, Δ-PPFE = annualised change in computerised upper-zone PPFE extent between scans.

Cohort	Dependent variable	Independent variable	Effect [l/year]	95% Confidence Interval [l/year]	p-value	Model R^2 value
IPF (n = 333)	FVC (l)	Δ-PPFE	-0.13	-0.18, -0.08	<0.0001	0.07
FHP (n = 78)	FVC (l)	Δ-PPFE	-0.08	-0.19, 0.02	0.10	0.03

Supplementary Table 9 Multivariable linear mixed-effects regression analyses between temporal FVC (l) measurements and Δ-PPFE. Multivariable linear mixed-effects regression analyses, adjusted for patient centre as a random intercept and for subject as a random slope and a random intercept, demonstrating relationships between FVC (l) change within a 6-month window of the baseline and follow-up CT scans of each patient and Δ-PPFE in the IPF cohort and in the FHP cohort. All models were adjusted for patient age at baseline, patient gender, smoking history (never/ever), baseline emphysema (absent/present), baseline FVC% predicted and Δ-PPFE. Models in the IPF cohort were also adjusted for antifibrotic treatment (never/ever). R^2 values shown are the marginal R^2 describing only the proportion of variance explained by fixed effects. IPF = idiopathic pulmonary fibrosis, FHP = fibrotic hypersensitivity pneumonitis, FVC = forced vital capacity, PPFE = pleuroparenchymal fibroelastosis, Δ-PPFE = annualised change in computerised upper-zone PPFE between scans.

Cohort	Dependent variable	Independent variable	Effect [l/year]	95% Confidence Interval [l/year]	p-value	Model R^2 value
IPF (n = 333)	FVC (l)	Δ-PPFE	-0.09	-0.13, -0.05	<0.0001	0.34
FHP (n = 78)	FVC (l)	Δ-PPFE	-0.04	-0.08, 0.004	0.08	0.61

Supplementary Table 10. **Non-imputed multivariable linear mixed-effects regression analyses between temporal FVC (l) measurements and Δ-PPFE.** Multivariable linear mixed-effects regression analyses, adjusted for patient centre as a random intercept and for subject as a random slope and a random intercept, demonstrating relationships between FVC (l) change within a 6-month window of the baseline and follow-up CT scans of each patient and Δ-PPFE in the IPF cohort and in the FHP cohort. All models were adjusted for patient age at baseline, patient gender, smoking history (never/ever), baseline emphysema presence (absent/present), baseline FVC% predicted and Δ-PPFE. Models in the IPF cohort were also adjusted for antifibrotic treatment (never/ever). In non-imputed models, patients with missing FVC% predicted within 3 months of baseline CT were excluded. R^2 values shown are the marginal R^2 describing only the proportion of variance explained by fixed effects. IPF = idiopathic pulmonary fibrosis, FHP = fibrotic hypersensitivity pneumonitis, FVC = forced vital capacity, PPFE = pleuroparenchymal fibroelastosis, Δ-PPFE = annualised change in computerised upper-zone PPFE between scans.

**Cohort**	**Dependent variable**	**Independent variable**	**Effect [l/year]**	**95% Confidence Interval [l/year]**	**p-value**	**Model R^2 value**
IPF (n = 290)	FVC (l)	Δ-PPFE	-0.09	-0.14, -0.05	0.00001	0.33
FHP (n = 67)	FVC (l)	Δ-PPFE	-0.04	-0.09, 0.005	0.070	0.60

Supplementary Table 11. Univariable Cox regression models showing mortality in the IPF cohort and in the FHP cohort. IPF = idiopathic pulmonary fibrosis, FHP = fibrotic hypersensitivity pneumonitis, DLco = diffusing capacity for carbon monoxide, PPFE = pleuroparenchymal fibroelastosis, AF = antifibrotic, ILD = interstitial lung disease, Δ-ILD = annualised change in ILD extent between scans, PPFE = pleuroparenchymal fibroelastosis, Δ-PPFE = annualised change in computerised upper-zone PPFE between scans, Δ-PPFE-adj = Δ-PPFE above scan noise.

Cohort	Variable	Hazard ratio	95% Confidence Interval	p-value	Model C-index
IPF	Age at baseline (years)	1.01	0.99, 1.03	0.26	0.60
	Male gender	1.38	0.98, 1.94	0.068	0.60
	Ever smoker	1.30	0.96, 1.76	0.096	0.60
	AF treatment (never/ever)	0.81	0.60, 1.08	0.15	0.60
	Baseline DLco% predicted	0.95	0.94, 0.96	< 0.0001	0.70
	Baseline FVC% predicted	0.98	0.97, 0.99	< 0.0001	0.66
	Baseline ILD extent (%)	1.04	1.03, 1.05	< 0.0001	0.68
	Δ-ILD (%/year)	1.01	1.00, 1.02	0.058	0.61
	Baseline emphysema presence	1.01	0.76, 1.35	0.93	0.59
	Baseline PPFE extent (%)	1.17	1.12, 1.22	< 0.0001	0.67
	Baseline clinically important PPFE presence (PPFE >2.5%)	2.52	1.92, 3.31	< 0.0001	0.67
	Δ-PPFE (%/year)	1.27	1.20, 1.35	< 0.0001	0.66
	Δ-PPFE-adj (%/year)	1.33	1.25, 1.42	< 0.0001	0.66
FHP	Age at baseline (years)	1.05	1.02, 1.08	0.004	0.69
	Male gender	1.33	0.73, 2.42	0.36	0.64
	Ever smoker	1.28	0.70, 2.34	0.43	0.64
	Baseline DLco% predicted	0.96	0.93, 0.99	0.006	0.74
	Baseline FVC% predicted	0.98	0.96, 1.00	0.11	0.67
	Baseline ILD extent (%)	1.06	1.04, 1.09	< 0.0001	0.77
	Δ-ILD (%/year)	1.04	0.98, 1.09	0.21	0.68
	Baseline emphysema presence	0.85	0.43, 1.67	0.63	0.64
	Baseline PPFE extent (%)	1.20	1.06, 1.36	0.003	0.73
	Baseline clinically important PPFE presence (PPFE >2.5%)	1.43	0.73, 2.81	0.30	0.67
	Δ-PPFE (%/year)	1.30	1.17, 1.44	< 0.0001	0.73
	Δ-PPFE-adj (%/year)	1.30	1.15, 1.46	< 0.0001	0.72

Supplementary Table 12: Association of Δ-PPFE with mortality in multivariable Cox regression models in the IPF cohort and in the FHP cohort. Models in all cohorts were adjusted for patient age, gender, smoking history (never/ever), baseline emphysema presence (absent/present), baseline DLco% predicted, baseline presence of clinically important PPFE, and Δ-PPFE. Models in the IPF cohort were additionally adjusted for antifibrotic treatment (never/ever). IPF = idiopathic pulmonary fibrosis, FHP = fibrotic hypersensitivity pneumonitis, DLco = diffusing capacity for carbon monoxide, PPFE = pleuroparenchymal fibroelastosis, AF = antifibrotic, Δ-PPFE = annualised change in computerised upper-lung PPFE between scans.

Cohort	Variable	Hazard ratio	95% Confidence Interval	p-value	Model C-index
IPF (n = 414)	Baseline age (years)	1.00	0.99, 1.02	0.72	0.75
	Male gender	1.53	1.05, 2.24	0.028	
	Ever smoker	1.13	0.80, 1.57	0.49	
	Baseline emphysema presence	0.98	0.70, 1.36	0.88	
	AF treatment (never/ever)	0.72	0.53, 0.96	0.027	
	Δ-ILD (%/year)	1.01	0.99, 1.02	0.22	
	Baseline clinically important PPFE (PPFE extent >2.5%)	1.80	1.33, 2.43	0.00015	
	Baseline DLco% predicted	0.96	0.94, 0.97	< 0.0001	
	Δ-PPFE (%/year)	1.20	1.13, 1.28	< 0.0001	
FHP (n = 98)	Baseline age (years)	1.06	1.02, 1.10	0.003	0.81
	Male gender	1.26	0.53, 2.98	0.59	
	Ever smoker	0.97	0.43, 2.19	0.95	
	Baseline emphysema presence	0.65	0.27, 1.57	0.33	
	Δ-ILD (%/year)	1.05	0.99, 1.12	0.12	
	Baseline clinically important PPFE (PPFE extent >2.5%)	1.32	0.56, 3.13	0.51	
	Baseline DLco% predicted	0.97	0.94, 1.00	0.028	
	Δ-PPFE (%/year)	1.21	1.07, 1.38	0.004	

Supplementary Table 13: Association of Δ-PPFE with mortality in non-imputed multivariable Cox regression models in the IPF cohort and in the FHP cohort. Models in all cohorts were adjusted for patient age, gender, smoking history (never/ever), baseline emphysema presence (absent/present), baseline DLco% predicted, baseline presence of clinically important PPFE, and Δ-PPFE. Models in the IPF cohort were additionally adjusted for antifibrotic treatment (never/ever). In non-imputed models, patients with missing DLco% predicted within 3 months of baseline CT were excluded. IPF = idiopathic pulmonary fibrosis, FHP = fibrotic hypersensitivity pneumonitis, DLco = diffusing capacity for carbon monoxide, PPFE = pleuroparenchymal fibroelastosis, AF = antifibrotic, Δ-PPFE = annualised change in computerised upper-lung PPFE between scans.

Cohort	Variable	Hazard ratio	95% Confidence Interval	p-value	Model C-index
IPF (n = 319)	Baseline age (years)	1.00	0.98, 1.02	0.93	0.75
	Male gender	1.59	1.04, 2.44	0.034	
	Ever smoker	1.21	0.84, 1.75	0.31	
	Baseline emphysema presence	1.04	0.72, 1.51	0.82	
	AF treatment (never/ever)	0.65	0.47, 0.90	0.008	
	Δ-ILD (%/year)	1.00	0.99, 1.02	0.63	
	Baseline clinically important PPFE (PPFE extent >2.5%)	1.73	1.24, 2.41	0.001	
	Baseline DLco% predicted	0.95	0.94, 0.97	< 0.0001	
	Δ-PPFE (%/year)	1.26	1.18, 1.35	< 0.0001	
FHP (n = 66)	Baseline age (years)	1.06	1.01, 1.12	0.020	0.83
	Male gender	0.82	0.31, 2.15	0.69	
	Ever smoker	2.13	0.71, 6.44	0.18	
	Baseline emphysema presence	1.08	0.42, 2.78	0.88	
	Δ-ILD (%/year)	1.10	1.01, 1.19	0.035	
	Baseline clinically important PPFE (PPFE extent >2.5%)	2.24	0.83, 6.09	0.11	
	Baseline DLco% predicted	0.96	0.93, 0.99	0.010	
	Δ-PPFE (%/year)	1.33	1.11, 1.59	0.002	

Supplementary Table 14: Multivariable Cox regression models in the IPF cohort and in the FHP cohort without adjustment for Δ-PPFE. Models in all cohorts were adjusted for patient age, gender, smoking history (never/ever), baseline emphysema presence (absent/present), baseline DLco% predicted, and baseline presence of clinically important PPFE. Models in the IPF cohort were additionally adjusted for antifibrotic treatment (never/ever). IPF = idiopathic pulmonary fibrosis, FHP = fibrotic hypersensitivity pneumonitis, DLco = diffusing capacity for carbon monoxide, PPFE = pleuroparenchymal fibroelastosis, AF = antifibrotic.

Cohort	Variable	Hazard ratio	95% Confidence Interval	p-value	Model C-index
IPF (n = 414)	Baseline age (years)	1.00	0.99, 1.02	0.78	0.74
	Male gender	1.34	0.92, 1.95	0.12	
	Ever smoker	1.26	0.90, 1.77	0.18	
	Baseline emphysema presence	0.83	0.59, 1.16	0.27	
	AF treatment (never/ever)	0.72	0.53, 0.97	0.031	
	Δ-ILD (%/year)	1.02	1.00, 1.03	0.031	
	Baseline clinically important PPFE (PPFE extent >2.5%)	1.87	1.39, 2.52	0.00005	
	Baseline DLco% predicted	0.95	0.94, 0.97	< 0.0001	
FHP (n = 98)	Baseline age (years)	1.06	1.02, 1.11	0.002	0.77
	Male gender	1.61	0.70, 3.69	0.26	
	Ever smoker	0.90	0.40, 2.03	0.80	
	Baseline emphysema presence	0.53	0.23, 1.24	0.14	
	Δ-ILD (%/year)	1.06	0.99, 1.13	0.09	
	Baseline clinically important PPFE (PPFE extent >2.5%)	1.84	0.86, 3.91	0.11	
	Baseline DLco% predicted	0.96	0.93, 0.99	0.016	

Supplementary Table 15. **Demographic data comparing IPF patients without clinically important PPFE at baseline and without progressive PPFE, patients with clinically important PPFE at baseline and without progressive PPFE, and patients with progressive PPFE.** Baseline demographic data, pulmonary function indices and disease severity metrics in IPF patients a) without clinically important PPFE at baseline and without progressive PPFE, b) with clinically important PPFE at baseline and without progressive PPFE, and c) with progressive PPFE. Clinically important PPFE at baseline was defined as baseline PPFE extent >2.5%. Progressive PPFE (i.e., longitudinal increase in PPFE above scan noise) was defined as Δ-PPFE >1.25%/year. Statistical tests were made to test for independence (categorical variables) and differences in means/medians (continuous variables) across the three groups. IPF = idiopathic pulmonary fibrosis, FVC = forced vital capacity, DLco = diffusing capacity for carbon monoxide, ILD = interstitial lung disease, PPFE = pleuroparenchymal fibroelastosis, Δ-PPFE = annualised change in computerised upper-zone PPFE between scans, Δ-PPFE-adj = Δ-PPFE above scan noise.

**Variable**	**No clinically important PPFE at baseline, no progressive PPFE**	**Clinically important PPFE at baseline, no progressive PPFE**	**Progressive PPFE**	**p-value**
IPF cohort:	(n = 256)	(n = 69)	(n = 89)	
Median baseline age in years (range)	68.5 (32.0 - 95.0)	69.0 (42.0 - 83.0)	70.0 (37.0 - 84.0)	0.95
Male / female	73.4% / 26.6%	84.1% / 15.9%	76.4% / 23.6%	0.19
Survival (alive / dead)	55.1% / 44.9%	30.4% / 69.6%	24.7% / 75.3%	<0.0001
Median years of follow-up (range)	3.0 (0.0 - 9.0)	1.7 (0.0 - 8.2)	1.2 (0.0 - 5.9)	<0.0001
Median years between CT scans (range)	1.2 (0.5 - 3.0)	1.1 (0.5 - 2.8)	1.0 (0.5 - 2.9)	0.002
Never / ever smokers	32.8% / 67.2%	29.0% / 71.0%	25.8% / 74.2%	0.44
Antifibrotic (never / ever)	28.9% / 71.1%	31.9% / 68.1%	34.8% / 65.2%	0.57
Baseline FVC% predicted	86.1 +/- 18.3	74.1 +/- 18.3	72.2 +/- 20.2	<0.0001
Baseline DLco% predicted	51.8 +/- 14.1	41.4 +/- 14.0	44.1 +/- 14.1	<0.0001
Baseline emphysema (absent/present)	27.3% / 72.7%	33.3% / 66.7%	46.1% / 53.9%	0.005
Baseline ILD extent (%)	36.0 +/- 11.5	45.3 +/- 12.8	42.8 +/- 11.3	<0.0001
Δ-ILD (%/year)	6.2 +/- 6.5	8.2 +/- 7.8	11.5 +/- 12.9	<0.0001
Baseline PPFE extent (%)	0.8 +/- 0.7	4.8 +/- 2.5	3.5 +/- 2.8	<0.0001
Δ-PPFE (%/year)	0.2 +/- 0.4	-0.5 +/- 1.5	3.5 +/- 2.7	<0.0001
Clinically important baseline PPFE prevalence	0.0%	100.0%	59.6%	<0.0001
Progressive PPFE prevalence	0.0%	0.0%	100.0%	<0.0001
Δ-PPFE-adj (progressive PPFE patients, %/year)	–	–	2.3 +/- 2.7	–

Supplementary Table 16. **Demographic data comparing FHP patients without clinically important PPFE at baseline and without progressive PPFE, patients with clinically important PPFE at baseline and without progressive PPFE, and patients with progressive PPFE.** Baseline demographic data, pulmonary function indices and disease severity metrics in FHP patients a) without clinically important PPFE at baseline and without progressive PPFE, b) with clinically important PPFE at baseline and without progressive PPFE, and c) with progressive PPFE. Clinically important PPFE at baseline was defined as baseline PPFE extent >2.5%. Progressive PPFE (i.e., longitudinal increase in PPFE above scan noise) was defined as Δ-PPFE >1.25%/year. Statistical tests were made to test for independence (categorical variables) and differences in means/medians (continuous variables) across the three groups. FHP = fibrotic hypersensitivity pneumonitis, FVC = forced vital capacity, DLco = diffusing capacity for carbon monoxide, ILD = interstitial lung disease, PPFE = pleuroparenchymal fibroelastosis, Δ-PPFE = annualised change in computerised upper-zone PPFE between scans, Δ-PPFE-adj = Δ-PPFE above scan noise.

Variable	No clinically important PPFE at baseline, no progressive PPFE	Clinically important PPFE at baseline, no progressive PPFE	Progressive PPFE	p-value
FHP cohort:	(n = 56)	(n = 17)	(n = 25)	
Median baseline age in years (range)	64.5 (28.0 - 85.0)	67.0 (40.0 - 81.0)	61.0 (41.0 - 82.0)	0.72
Male / female	37.5% / 62.5%	35.3% / 64.7%	40.0% / 60.0%	0.95
Survival (alive / dead)	64.3% / 35.7%	58.8% / 41.2%	28.0% / 72.0%	0.009
Median years of follow-up (range)	3.3 (0.4 - 10.4)	2.6 (0.0 - 12.0)	1.8 (0.0 - 6.0)	0.0001
Never / ever smokers	48.2% / 51.8%	41.2% / 58.8%	60.0% / 40.0%	0.45
Baseline FVC% predicted	68.3 +/- 21.3	62.8 +/- 16.0	56.2 +/- 15.3	0.066
Baseline DLco% predicted	53.1 +/- 16.4	50.7 +/- 19.4	44.7 +/- 15.5	0.23
Baseline emphysema (absent/present)	66.1% / 33.9%	64.7% / 35.3%	80.0% / 20.0%	0.41
Baseline ILD extent (%)	28.8 +/- 13.3	39.9 +/- 12.7	39.0 +/- 12.8	0.0007
Δ-ILD (%/year)	2.8 +/- 5.7	4.5 +/- 4.7	6.3 +/- 5.0	0.027
Median years between CT scans (range)	1.2 (0.5 - 2.8)	1.2 (0.7 - 2.9)	1.0 (0.5 - 2.8)	0.28
Baseline PPFE extent (%)	0.7 +/- 0.6	4.2 +/- 1.9	3.1 +/- 3.0	<0.0001
Δ-PPFE (%/year)	0.0 +/- 0.5	-0.6 +/- 0.9	3.6 +/- 3.3	<0.0001
Clinically important baseline PPFE prevalence	0.0%	100.0%	36.0%	<0.0001
Progressive PPFE prevalence	0.0%	0.0%	100.0%	<0.0001
Δ-PPFE-adj (progressive PPFE patients, %/year)	–	–	2.4 +/- 3.3	–

Supplementary Table 17: Association of Δ-PPFE-adj with mortality in non-imputed multivariable Cox regression models in the IPF cohort and in the FHP cohort. Models in all cohorts were adjusted for patient age, gender, smoking history (never/ever), baseline emphysema presence (absent/present), baseline DLco% predicted, and Δ-PPFE-adj. Models in the IPF cohort were additionally adjusted for antifibrotic treatment (never/ever). In non-imputed models, patients with missing DLco% predicted within 3 months of baseline CT were excluded. IPF = idiopathic pulmonary fibrosis, FHP = fibrotic hypersensitivity pneumonitis, DLco = diffusing capacity for carbon monoxide, PPFE = pleuroparenchymal fibroelastosis, AF = antifibrotic, Δ-PPFE-adj = annualised change in computerised upper-zone PPFE above scan noise.

Cohort	Variable	Hazard ratio	95% Confidence Interval	p-value	Model C-index
IPF (n = 319)	Baseline age (years)	1.00	0.98, 1.02	0.86	0.75
	Male gender	1.50	0.97, 2.30	0.067	
	Ever smoker	1.32	0.91, 1.93	0.15	
	Baseline emphysema presence	0.99	0.68, 1.43	0.95	
	AF treatment (never/ever)	0.65	0.47, 0.90	0.009	
	Δ-ILD (%/year)	1.00	0.99, 1.02	0.54	
	Baseline clinically important PPFE (PPFE extent >2.5%)	1.67	1.19, 2.33	0.003	
	Baseline DLco% predicted	0.96	0.94, 0.97	< 0.00001	
	Δ-PPFE-adj (%/year)	1.32	1.22, 1.43	< 0.00001	
FHP (n = 66)	Baseline age (years)	1.05	1.00, 1.11	0.037	0.81
	Male gender	0.88	0.34, 2.28	0.79	
	Ever smoker	2.16	0.72, 6.51	0.17	
	Baseline emphysema presence	0.91	0.36, 2.33	0.84	
	Δ-ILD (%/year)	1.10	1.01, 1.19	0.028	
	Baseline clinically important PPFE (PPFE extent >2.5%)	2.12	0.79, 5.67	0.13	
	Baseline DLco% predicted	0.96	0.93, 0.99	0.008	
	Δ-PPFE-adj (%/year)	1.30	1.07, 1.57	0.008	

Supplementary Table 18: Association of Δ-PPFE-adj with mortality in multivariable Cox regression models in the IPF cohort and in the FHP cohort, without adjustment for baseline presence of clinically important PPFE. Models in all cohorts were adjusted for patient age, gender, smoking history (never/ever), baseline emphysema presence (absent/present), baseline DLco% predicted, and Δ-PPFE. Models in the IPF cohort were additionally adjusted for antifibrotic treatment (never/ever). IPF = idiopathic pulmonary fibrosis, FHP = fibrotic hypersensitivity pneumonitis, DLco = diffusing capacity for carbon monoxide, PPFE = pleuroparenchymal fibroelastosis, AF = antifibrotic, Δ-PPFE-adj=annualised change in computerised upper-zone PPFE between scans above scan noise.

Cohort	Variable	Hazard ratio	95% Confidence Interval	p-value	Model C-index
IPF (n = 414)	Baseline age (years)	1.00	0.99, 1.02	0.58	0.74
	Male gender	1.48	1.00, 2.17	0.047	
	Ever smoker	1.21	0.86, 1.71	0.27	
	Baseline emphysema presence	0.85	0.61, 1.18	0.33	
	AF treatment (never/ever)	0.72	0.53, 0.97	0.030	
	Δ-ILD (%/year)	1.01	1.00, 1.03	0.094	
	Baseline DLco% predicted	0.95	0.94, 0.96	<0.00001	
	Δ-PPFE-adj (%/year)	1.28	1.18, 1.37	<0.00001	
FHP (n = 98)	Baseline age (years)	1.06	1.02, 1.10	0.004	0.79
	Male gender	1.35	0.58, 3.13	0.47	
	Ever smoker	0.94	0.42, 2.09	0.87	
	Baseline emphysema presence	0.66	0.28, 1.51	0.31	
	Δ-ILD (%/year)	1.05*	0.99, 1.12	0.11	
	Baseline DLco% predicted	0.96	0.94, 0.99	0.020	
	Δ-PPFE-adj (%/year)	1.23	1.07, 1.40	0.004	

Supplementary Table 19: Association of Δ-PPFE-adj with mortality in multivariable Cox regression models in the IPF cohort (n = 414), with varying threshold of scan noise. Models were adjusted for patient age, gender, smoking history (never/ever), baseline emphysema presence (absent/present), antifibrotic treatment (never/ever), baseline DLco% predicted, baseline presence of clinically important PPFE, and Δ-PPFE-adj. The threshold of scan noise was varied between models. IPF = idiopathic pulmonary fibrosis, FHP = fibrotic hypersensitivity pneumonitis, DLco = diffusing capacity for carbon monoxide, PPFE = pleuroparenchymal fibroelastosis, AF = antifibrotic, Δ-PPFE-adj=annualised change in computerised upper-zone PPFE between scans above scan noise.

Threshold used to determine scan noise (%/year)	Variable	Hazard ratio	95% Confidence Interval	p-value	Model C-index
0.5	Baseline age (years)	1.00	0.99, 1.02	0.75	0.75
	Male gender	1.48	1.01, 2.16	0.046	
	Ever smoker	1.19	0.85, 1.66	0.32	
	Baseline emphysema presence	0.95	0.68, 1.33	0.77	
	AF treatment (never/ever)	0.72	0.53, 0.97	0.030	
	Δ-ILD (%/year)	1.01	1.00, 1.02	0.20	
	Baseline clinically important PPFE (PPFE extent >2.5%)	1.68	1.24, 2.27	0.0009	
	Baseline DLco% predicted	0.96	0.94, 0.97	<0.00001	
	Δ-PPFE-adj (%/year)	1.23	1.15, 1.32	<0.00001	
0.75	Baseline age (years)	1.00	0.99, 1.02	0.76	0.75
	Male gender	1.47	1.00, 2.15	0.048	
	Ever smoker	1.20	0.85, 1.68	0.29	
	Baseline emphysema presence	0.94	0.67, 1.32	0.74	
	AF treatment (never/ever)	0.72	0.54, 0.97	0.031	
	Δ-ILD (%/year)	1.01	1.00, 1.02	0.18	
	Baseline clinically important PPFE (PPFE extent >2.5%)	1.69	1.25, 2.29	0.0008	
	Baseline DLco% predicted	0.96	0.94, 0.97	<0.00001	
	Δ-PPFE-adj (%/year)	1.24	1.15, 1.33	<0.00001	
1.0	Baseline age (years)	1.00	0.99, 1.02	0.75	0.75
	Male gender	1.46	1.00, 2.14	0.051	
	Ever smoker	1.21	0.86, 1.69	0.27	
	Baseline emphysema presence	0.94	0.67, 1.31	0.70	
	AF treatment (never/ever)	0.72	0.54, 0.97	0.032	
	Δ-ILD (%/year)	1.01	1.00, 1.02	0.16	
	Baseline clinically important PPFE (PPFE extent >2.5%)	1.70	1.26, 2.30	0.0006	
	Baseline DLco% predicted	0.96	0.94, 0.97	<0.00001	
	Δ-PPFE-adj (%/year)	1.24	1.15, 1.33	<0.00001	
1.5	Baseline age (years)	1.00	0.99, 1.02	0.72	0.75
	Male gender	1.44	0.98, 2.11	0.061	
	Ever smoker	1.23	0.88, 1.72	0.23	
	Baseline emphysema presence	0.92	0.66, 1.29	0.64	
	AF treatment (never/ever)	0.73	0.54, 0.98	0.036	
	Δ-ILD (%/year)	1.01	1.00, 1.03	0.13	
	Baseline clinically important PPFE (PPFE extent >2.5%)	1.73	1.28, 2.33	0.0005	
	Baseline DLco% predicted	0.96	0.94, 0.97	0.00001	
	Δ-PPFE-adj (%/year)	1.25	1.16, 1.35	0.00001	

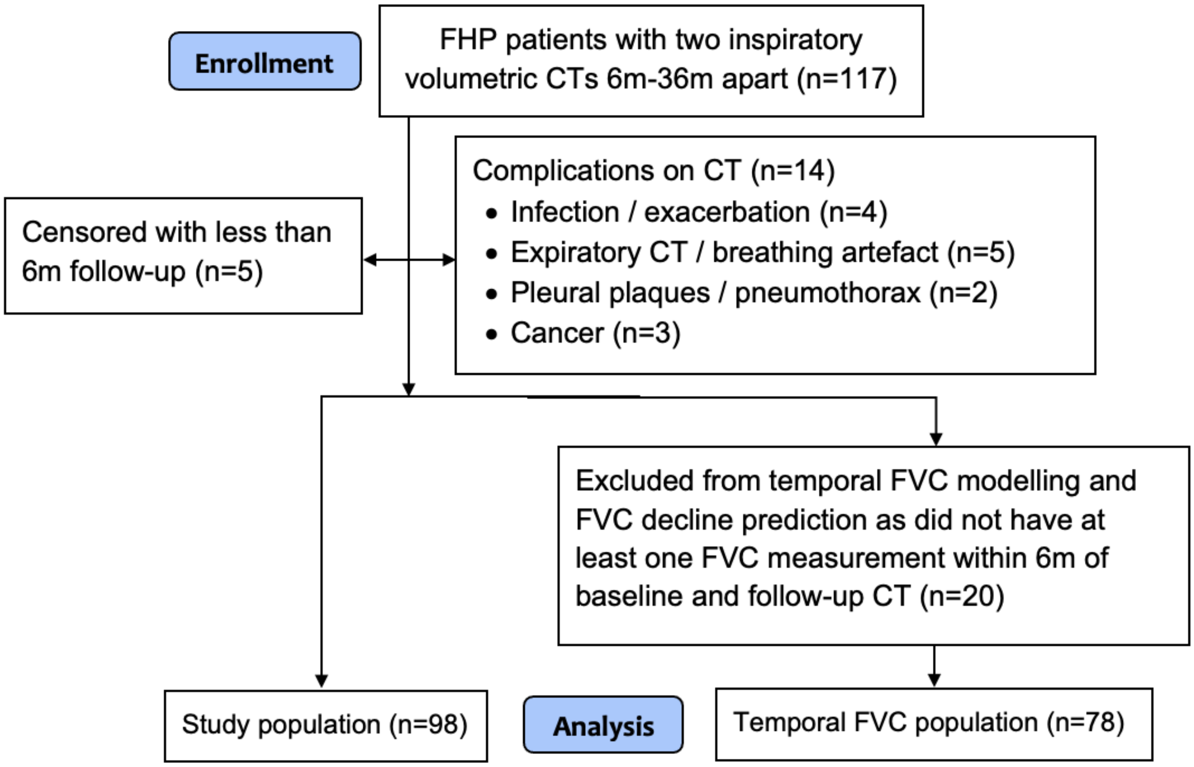

Supplementary Figure 1. **CONSORT diagram showing patient exclusions for FHP patients in the study.** CONSORT flow diagrams for all FHP patients in the study. FHP = fibrotic hypersensitivity pneumonitis, CT = computed tomography, FVC = forced vital capacity.

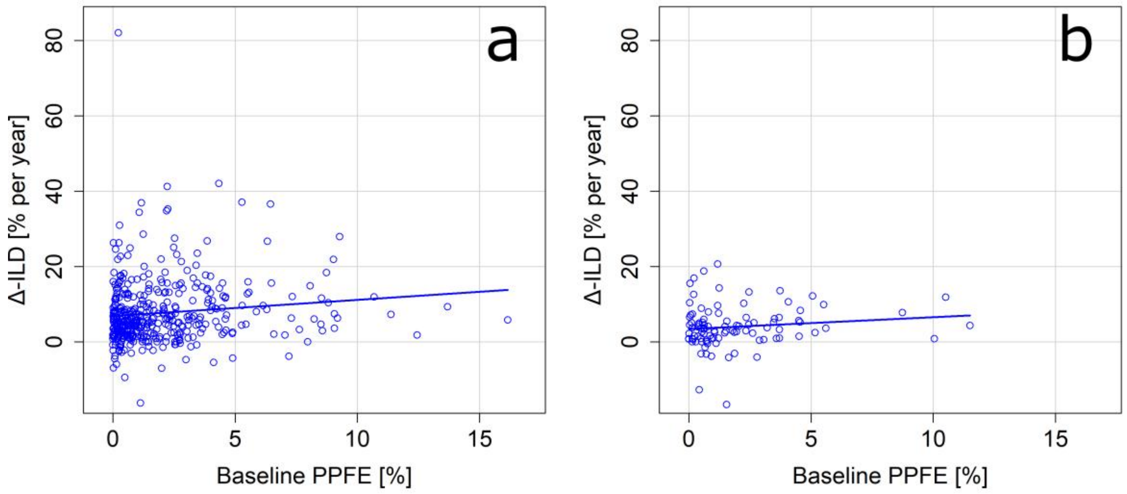

Supplementary Figure 2. **Association between Δ-ILD and baseline PPFE in the IPF cohort (a) and the FHP cohort (b).** a) Relationship between Δ-ILD and baseline PPFE in the IPF cohort, with a line of best fit shown (effect=0.36 %/year, 95% CI= 0.01–0.70 %/year, p=0.043, R^2=0.01). b) Relationship between Δ-ILD and baseline PPFE in the FHP cohort, with a line of best fit shown (effect=0.32 %/year, 95% CI= -0.17–0.80 %/year, p=0.20, R^2=0.02). ILD = interstitial lung disease, Δ-ILD = annualised change in ILD extent between scans, PPFE = pleuroparenchymal fibroelastosis, baseline PPFE = baseline computerised upper-zone PPFE extent, IPF = idiopathic pulmonary fibrosis, FHP = fibrotic hypersensitivity pneumonitis.

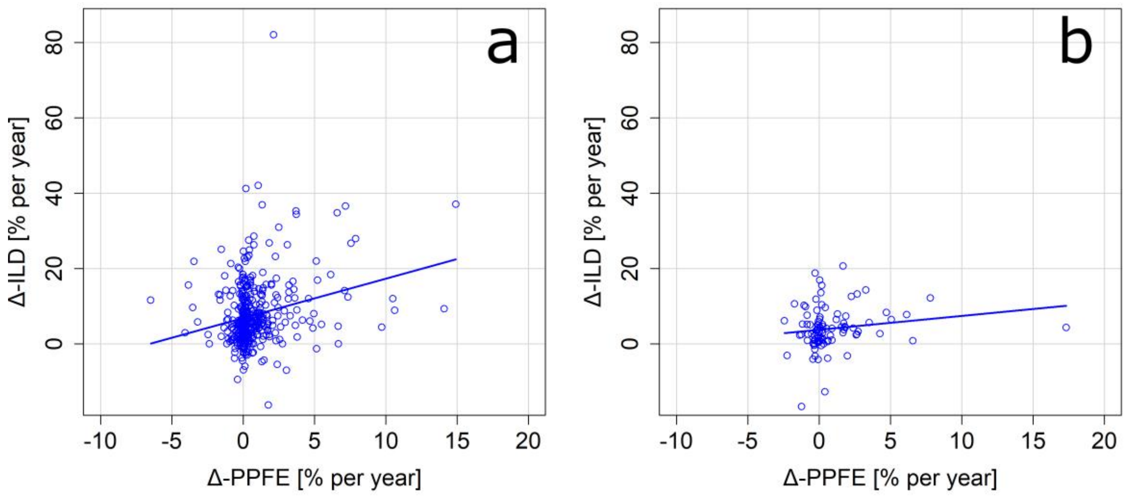

Supplementary Figure 3: Association between Δ-ILD and Δ-PPFE in the IPF cohort (a) and FHP cohort (b). a) Relationship between Δ-ILD and Δ-PPFE in the IPF cohort, with a line of best fit shown (effect=1.05 %/year, 95% CI=0.66–1.44 %/year, p<0.0001, R^2=0.06). b) Relationship between Δ-ILD and Δ-PPFE in the FHP cohort, with a line of best fit shown (effect=0.37 %/year, 95% CI= -0.08–0.82 %/year, p=0.11, R^2=0.03). IPF = idiopathic pulmonary fibrosis, FHP = fibrotic hypersensitivity pneumonitis, ILD = interstitial lung disease, Δ-ILD = annualised change in ILD extent between CT scans, PPFE = pleuroparenchymal fibroelastosis, Δ-PPFE = annualised change in computerised upper-zone PPFE between CT scans.

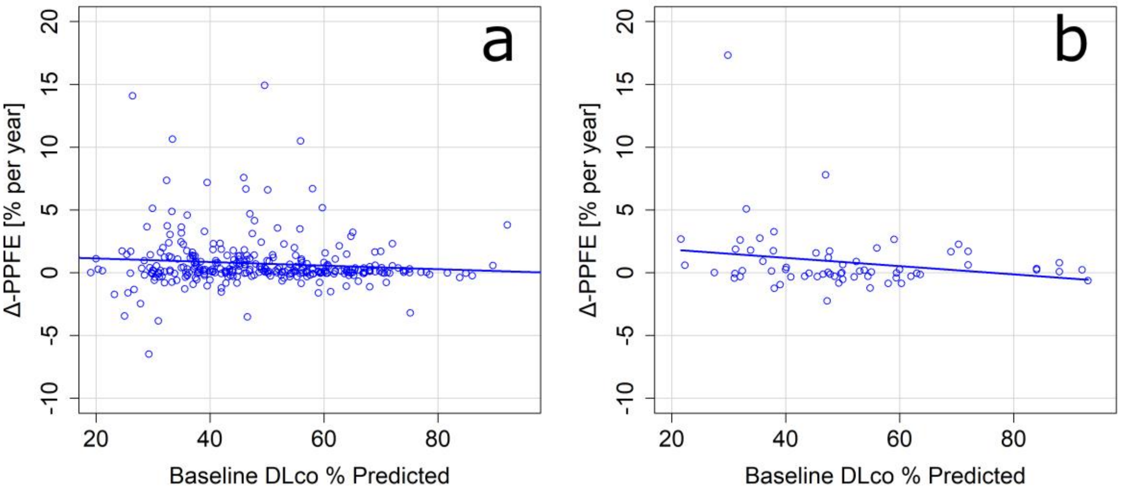

Supplementary Figure 4. **Association between Δ-PPFE and baseline DLco in the IPF cohort (a) and the FHP cohort (b).** a) Relationship between Δ-PPFE and baseline DLco% predicted in the IPF cohort, with a line of best fit shown (effect= -0.01 %/year, 95% CI= -0.03–0.0001 %/year, p=0.051, R^2=0.01). b) Relationship between Δ-PPFE and baseline DLco% predicted in the FHP cohort, with a line of best fit shown (effect= -0.03 %/year, 95% CI= -0.07–0.003 %/year, p=0.076, R^2=0.05). DLco = diffusing capacity for carbon monoxide, PPFE = pleuroparenchymal fibroelastosis, Δ-PPFE = annualised change in computerised upper-zone PPFE between scans, IPF = idiopathic pulmonary fibrosis, FHP = fibrotic hypersensitivity pneumonitis.

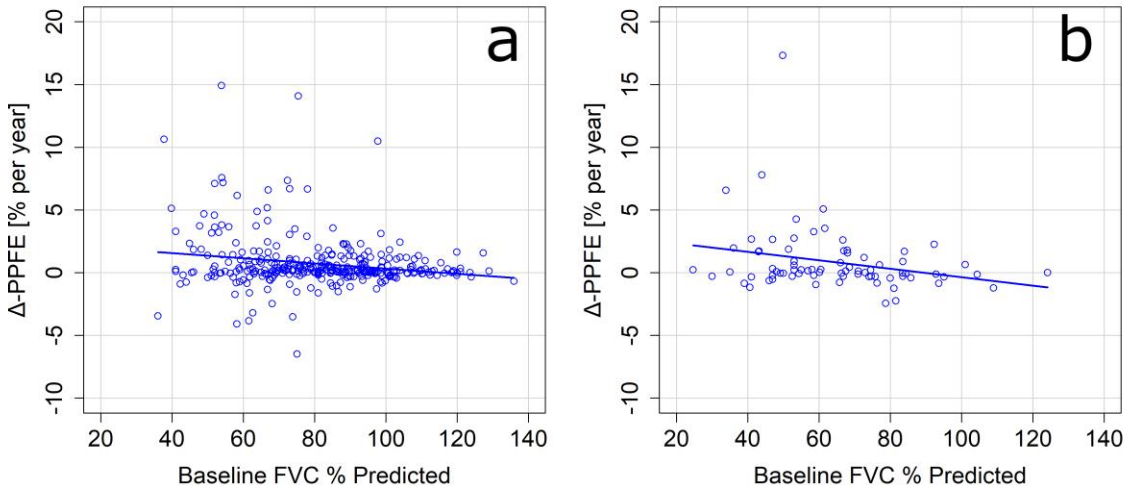

Supplementary Figure 5. **Association between Δ-PPFE and baseline FVC in the IPF cohort (a) and the FHP cohort (b).** a) Relationship between Δ-PPFE and baseline FVC% predicted in the IPF cohort, with a line of best fit shown (effect= -0.02 %/year, 95% CI= -0.03– -0.01 %/year, p=0.0003, R^2=0.04). b) Relationship between Δ-PPFE and baseline FVC% predicted in the FHP cohort, with a line of best fit shown (effect= -0.03 %/year, 95% CI= -0.06– -0.005 %/year, p=0.023, R^2=0.07). FVC = forced vital capacity, PPFE = pleuroparenchymal fibroelastosis, Δ-PPFE = annualised change in computerised upper-zone PPFE between scans, IPF = idiopathic pulmonary fibrosis, FHP = fibrotic hypersensitivity pneumonitis.

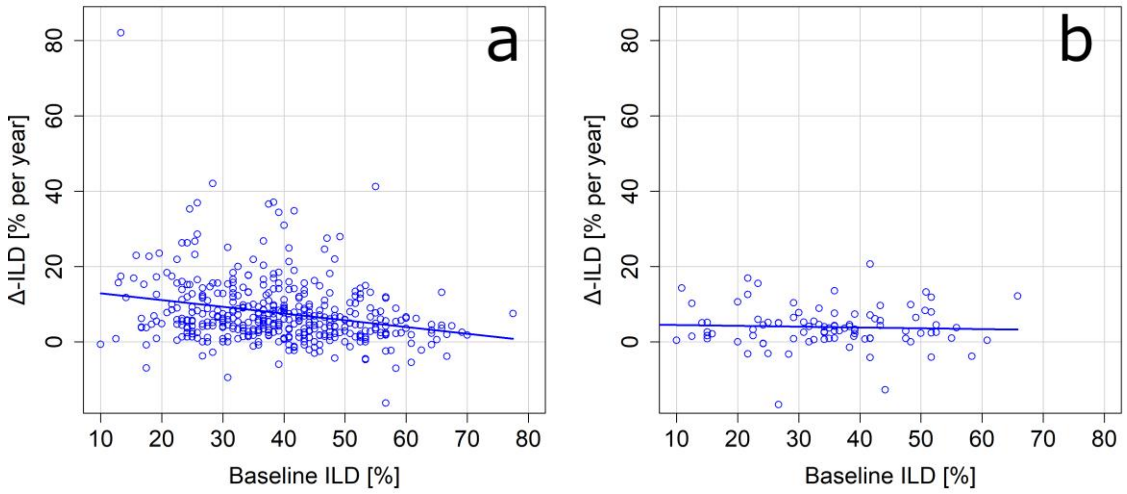

Supplementary Figure 6. **Association between Δ-ILD and baseline ILD extent in the IPF cohort (a) and the FHP cohort (b).** a) Relationship between Δ-ILD and baseline ILD extent in the IPF cohort, with a line of best fit shown (effect= -0.15 %/year, 95% CI= -0.22– -0.09 %/year, p<0.0001, R^2=0.05). b) Relationship between Δ-ILD and baseline ILD extent in the FHP cohort, with a line of best fit shown (effect= -0.02 %/year, 95% CI= -0.10–0.06 %/year, p=0.59, R^2 = 0.003). ILD = interstitial lung disease, Δ-ILD = annualised change in ILD extent between scans, PPFE = pleuroparenchymal fibroelastosis, Δ-PPFE = annualised change in computerised upper-zone PPFE between scans, IPF = idiopathic pulmonary fibrosis, FHP = fibrotic hypersensitivity pneumonitis.

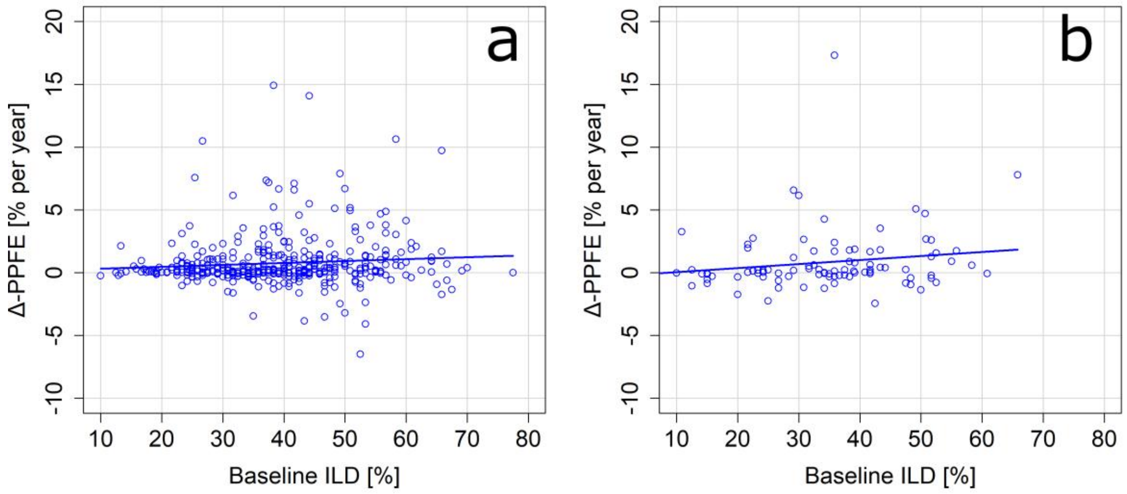

Supplementary Figure 7. **Association between Δ-PPFE and baseline ILD extent in the IPF cohort (a) and the FHP cohort (b).** Relationship between Δ-PPFE and baseline ILD extent in the IPF cohort, with a line of best fit shown (effect=0.02 %/year, 95% CI= -0.001–0.03 %/year, p=0.064, R^2=0.01). b) Relationship between Δ-PPFE and baseline ILD extent in the FHP cohort, with a line of best fit shown (effect=0.03 %/year, 95% CI= -0.002–0.07 %/year, p=0.063, R^2=0.04). ILD = interstitial lung disease, PPFE = pleuroparenchymal fibroelastosis, Δ-PPFE = annualised change in computerised upper-zone PPFE between scans, IPF = idiopathic pulmonary fibrosis, FHP = fibrotic hypersensitivity pneumonitis.

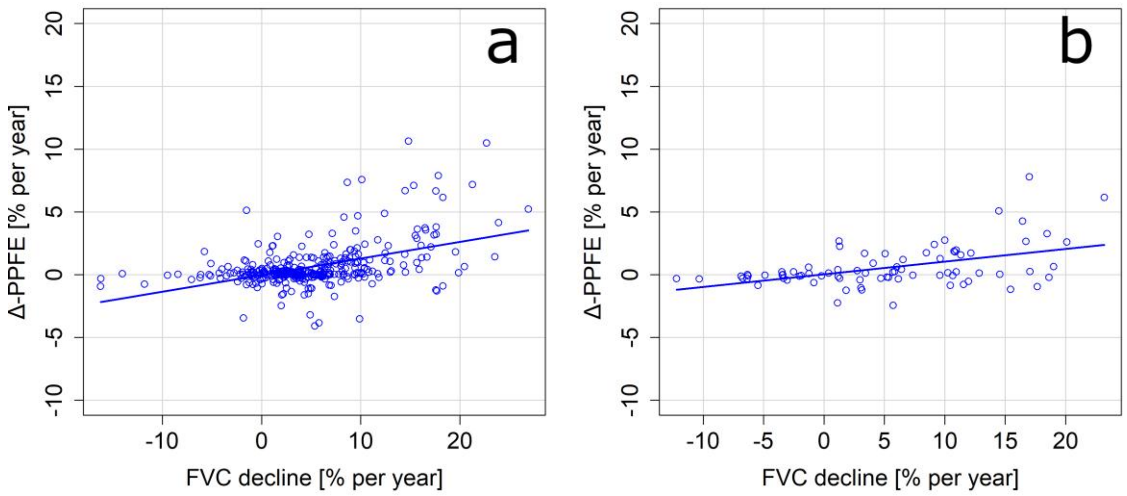

Supplementary Figure 8. **Association between Δ-PPFE and FVC decline per year in the IPF cohort (a) and the FHP cohort (b).** Relationship between Δ-PPFE and FVC decline per year in the IPF cohort, with a line of best fit shown (effect=0.13 %/year, 95% CI= 0.11–0.16 %/year, $p<0.0001$, $R^2=0.22$). b) Relationship between Δ-PPFE and baseline ILD extent in the FHP cohort, with a line of best fit shown (effect=0.10 %/year, 95% CI= 0.06–0.14 %/year, $p<0.0001$, $R^2=0.23$). ILD = interstitial lung disease, PPFE = pleuroparenchymal fibroelastosis, Δ-PPFE = annualised change in computerised upper-zone PPFE between scans, FVC = forced vital capacity, IPF = idiopathic pulmonary fibrosis, FHP = fibrotic hypersensitivity pneumonitis.

Supplementary References

1 Graham JW, Olchowski AE, Gilreath TD. How many imputations are really needed? Some practical clarifications of multiple imputation theory. *Prevention Science* 2007; **8**: 206–213.

2 Gudmundsson E, Zhao A, Mogulkoc N, *et al.* Pleuroparenchymal fibroelastosis in idiopathic pulmonary fibrosis: Survival analysis using visual and computer-based computed tomography assessment. *EClinicalMedicine* 2021; **38**: 101009.
